# High expression of lncRNA *PVT1* independently predicts poor overall survival in patients with primary uveal melanoma

**DOI:** 10.1371/journal.pone.0189675

**Published:** 2017-12-15

**Authors:** Haiming Xu, Jingwen Gong, Hui Liu

**Affiliations:** Department of Ophthalmology, Zhejiang Provincial People's Hospital, People's Hospital of Hangzhou Medical College, Hangzhou, Zhejiang, China; University of Alabama at Birmingham, UNITED STATES

## Abstract

The plasmacytoma variant translocation 1 gene (*PVT1*) plays an oncogenic role in the initiation and progression of multiple cancers. In this study, by using deep-sequencing data and follow-up data in the Cancer Genome Atlas-Uveal melanomas (TCGA-UVM), we assessed the association between the expression of *PVT1* and clinicopathological characteristics of patients with uveal melanoma, the mechanism of its dysregulation and its prognostic value. Results showed that high *PVT1* expression group had a higher proportion of epithelioid cell dominant disease (a more malignant histological subtype than spindle cell dominant disease) and more cases of extrascleral extension (a risk factor for metastasis) compared with the low *PVT1* expression group. 61 out of 80 cases (76.3%) of primary uveal melanoma had *PVT1* amplification in TCGA-UVM. In addition, *PVT1* expression was strongly and negatively correlated with its methylation status (Pearson's r = -0.712, Spearman’s r = -0.806). By performing univariate and multivariate analysis, we found that high *PVT1* expression was an independent predictor of poor OS in patients with uveal melanoma (HR: 12.015, 95%CI: 1.854–77.876, *p* = 0.009). Based on these findings, we infer that *PVT1* expression is modulated by both DNA amplification and methylation and its expression might serve as a valuable and specific prognostic biomarker in terms of OS in uveal melanoma.

## Introduction

Long noncoding RNAs (lncRNAs) are a class of RNA that are longer than 200 nucleotides and do not code for proteins [1]. Previous studies found that this class of RNAs play a pivotal role in regulating gene expression at both transcriptional and post-transcriptional levels [1]. Uveal melanoma, which is also known as ocular melanoma is the most common primary intraocular cancer in adults. Some recent studies found that dysregulated lncRNAs are involved in the pathological development of uveal melanoma [2, 3]. For example, hypermethylated in cancer 1 (HIC1) can induce uveal melanoma progression by activating lncRNA-numb [4]. LncRNA CASC15-New-Transcript 1(*CANT1*) acts as a necessary suppressor of uveal melanoma via triggering the expression of lncRNA *JPX* and *FTX* and subsequently inducing the expression of lncRNA *XIST* [5]. HOXA11-AS can increase uveal melanoma cell growth and invasion by interacting with enhancer of zeste homolog 2 (EZH2) to suppress its target p21 protein expression and by sponging miR-124 [3].

The plasmacytoma variant translocation 1 gene (*PVT1*) has been demonstrated as an oncogenic lncRNA in multiple cancers, including ovarian cancer [6], breast cancer [7], prostate cancer [8], cervical cancer [9], gastric cancer [10] and non-small cell lung cancer [11]. In gastric cancer, high *PVT1* expression is an independent prognostic marker for poor overall survival (OS) and disease-free survival (DFS) [10].

*PVT1* overexpression promotes melanoma cells proliferation, cell cycle progression, and migration [12]. Mechanistically, *PVT1* directly sponges miR-26b, which had been verified as a tumor suppressor in melanoma [13]. These findings suggest that *PVT1* may also act as an oncogene in melanoma. However, its association with uveal melanoma, as well as its prognostic value and the mechanism of its dysregulation in uveal melanoma have not been explored. In this study, by using deep-sequencing data and follow-up data in the Cancer Genome Atlas-Uveal melanomas (TCGA-UVM), we found that *PVT1* expression is modulated by both DNA amplification and methylation and its high expression independently predicts poor OS in patients with primary uveal melanoma.

## Materials and methods

### Data mining in the Cancer Genome Atlas-Uveal melanomas (UVM)

The level 3 data of patients with primary uveal melanoma in TCGA-UVM or with primary skin melanoma in TCGA-SKCM were downloaded by using the UCSC Xena browser (https://xenabrowser.net). Heatmap showing *PVT1* copy number alterations, RNA expression and DNA methylation (450k) was generated. Regression analysis of the correlation between *PVT1* RNA expression and its DNA methylation was examined by using cBioPortal for Cancer Genomics (http://www.cbioportal.org/) [14, 15].

Kaplan-Meier curves of overall survival (OS) were generated by GraphPad Prism v6.0 (GraphPad Software Inc.). Patients were grouped according to median *PVT1* expression or median *PVT1* DNA methylation.

### Statistical analysis

Statistical analysis was performed by using SPSS 19.0 (SPSS Inc.) and GraphPad Prism v6.0. Comparison of the clinicopathological features between high and low *PVT1* expression groups was performed using χ^2^ tests. Log-rank test was performed to assess the difference between the survival curves. Prognostic values were analyzed by univariate and multivariate Cox regression models. Welch’s t-test was conducted to compare *PVT1* expression between different copy number alterations. *P* < 0.05 was considered to be statistically significant.

## Results

### High expression of lncRNA *PVT1* is associated with malignant behaviors of uveal melanoma

The association between *PVT1* expression and the clinicopathological parameters was summarized in Table 1. Compared with the low *PVT1* expression group, the high *PVT1* expression was associated with older age (66.80 ± 11.55 *vs*. 56.50 ± 14.36, *p* = 0.0007), a higher proportion of epithelioid cell dominant disease (22/40 *vs*. 12/40, *p* = 0.024), more cases of distant metastasis (4/28 *vs*. 0/27, p = 0.043) and extrascleral extension (6/37 *vs*. 1/38, *p* = 0.043) and a higher death rate (20/40 *vs*. 3/40, *p*<0.0001) (Table 1).

**Table 1:** The association between *PVT1* expression and the clinicopathological parameters in patients with primary uveal melanoma in TCGA.

Parameters		*PVT1* expression RNAseq		χ^2^	*p* Value
		High (N = 40)	Low (N = 40)		
Age (Mean ± SD)		66.80 ± 11.55	56.50 ± 14.36		0.0007
Gender	Female	18	17	0.051	0.82
	Male	22	23		
Histological type	Epithelioid cell dominant	22	12	5.12	0.024
	Spindle Cell dominant	18	28		
Pathological T	II	6	8	0.35	0.56
	III/IV	34	32		
Pathological N	N0	25	27	N.A.	N.A
	NX/null	15	13		
Pathological M	M0	24	27	4.16	0.041
	M1+	4	0		
	MX/null	12	13		
Pathological stages	II	18	21	0.32	0.57
	III/IV	21	19		
	Null	1	0		
Tumor diameter (mm)	≤16	16	18	0.13	0.72
	>16	23	22		
	Null	1	0		
Tumor thickness (mm)	≤10	15	22	2.46	0.12
	>10	25	18		
Extrascleral extension	No	31	37	4.09	0.043
	Yes	6	1		
	Null	3	2		
Living Status	Living	20	37	17.64	<0.0001
	Dead	20	3		

Extrascleral extension: extension occurring outside the sclera of the orbit; M1+: M1a/M1b/M1c; NX: Nearby (regional) lymph nodes cannot be assessed; MX: Metastasis cannot be measured; Null: data was not available; N/A.: not applicable.

### The expression of *PVT1* is modulated by DNA amplification and methylation in uveal melanoma

**Fig 1:**
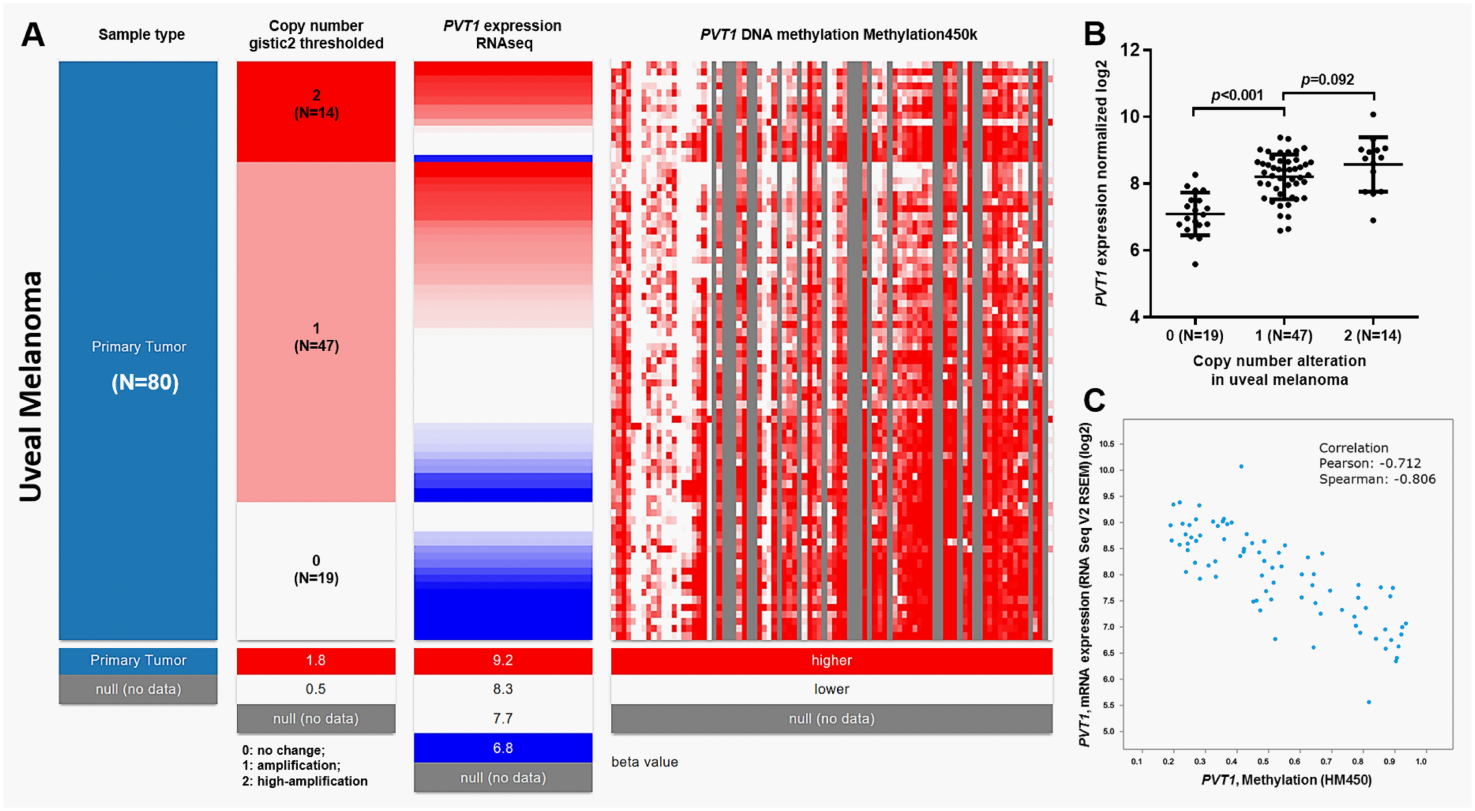
The expression of *PVT1* is modulated by DNA amplification and methylation in uveal melanoma. **A**. Heatmap of *PVT1* copy number alterations, *PVT1* RNA expression and *PVT1* DNA methylation in 80 cases of primary uveal melanoma. **B**. Bar chart of *PVT1* expression in high-amplification (2), amplification (1) and no change (0) groups. **C**. Regression analysis of the correlation between *PVT1* RNA expression and DNA methylation.

By using the deep sequencing data in TCGA, we tried to explore the mechanisms of *PVT1* dysregulation in uveal melanoma. Gene-level thresholded GISTIC2-processed copy-number data, which defines genetic changes as homozygous deletion (-2), heterozygous loss (-1), copy-neutral (0), low-level copy gain (+1), high-level amplification (+2) were downloaded from the Xena browser. Among the 80 cases of primary uveal melanoma, 14 cases (17.5%) had *PVT1* high-amplification (+2) and 47 cases (58.8%) had amplification (+1) (Fig 1A). The amplification was associated with significantly higher expression of *PVT1* RNA (Fig 1B). However, no significant difference was observed between the +2 and +1 group (Fig 1B). Then, we characterized the correlation between *PVT1* expression and its DNA methylation (Fig 1A and 1C). Heatmap and following regression analysis revealed a strong negative correlation between *PVT1* expression and DNA methylation (Pearson's r = -0.712, Spearman’s r = -0.806) (Fig 1A and 1C).

### High expression of *PVT1* independently predicts poor OS in patients with primary uveal melanoma

**Fig 2:**
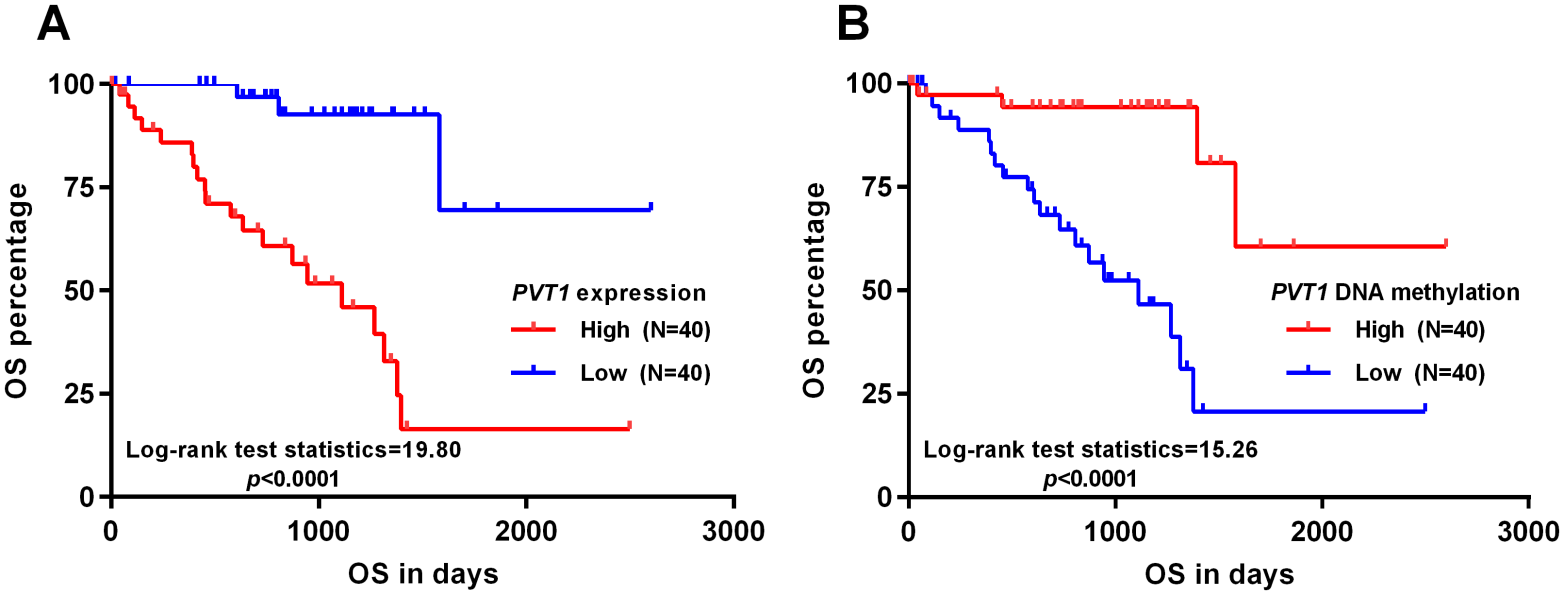
High expression of *PVT1* is associated with poor OS in patients with primary uveal melanoma. **A-B**. Kaplan-Meier curves of OS in uveal melanoma patients grouped by the median *PVT1* expression (A) or *PVT1* methylation (B).

Since the expression of *PVT1* was associated with malignant behaviors of uveal melanoma, we determine to assess its prognostic value. By generating Kaplan-Meier curves of OS, we found that high *PVT1* expression was associated with significantly shorter OS (*p*<0.0001) (Fig 2A). Interestingly, as a mechanism of *PVT1* dysregulation, low *PVT1* DNA methylation was also associated with unfavorable OS (*p*<0.0001) (Fig 2B). In univariate analysis, we found that epithelioid cell dominant uveal melanoma, extrascleral extension, high *PVT1* expression and low *PVT1* DNA methylation were associated with unfavorable OS (Table 2). Multivariate analysis showed that older age (>60) (HR: 2.599, 95%CI: 1.049–6.437, *p* = 0.039), epithelioid cell dominant uveal melanoma (HR: 4.385, 95%CI: 1.514–12.703, *p* = 0.006) and high *PVT1* expression (HR: 12.015, 95%CI: 1.854–77.876, *p* = 0.009) were independent predictors for poor OS (Table 2).

**Table 2:** Univariate and multivariate analyses of OS in patients with primary uveal melanoma.

Parameters	Univariate analysis				Multivariate analysis			
	*p*	HR	95%CI (lower/upper)		*p*	HR	95%CI (lower/upper)	
Age > 60 *vs*.≤ 60	0.080	2.123	0.914	4.933	0.039	2.599	1.049	6.437
Female *vs*. Male	0.325	0.649	0.274	1.536				
Histological type Epithelioid cell dominant *vs*. Spindle Cell dominant	0.001	4.551	1.814	11.418	0.006	4.385	1.514	12.703
Pathological stage III/IV *vs*. II	0.358	1.504	0.630	3.589				
Tumor diameter (mm) >16 *vs*. ≤16	0.192	1.831	0.738	4.541				
Tumor thickness (mm) >10 *vs*. ≤10	0.106	2.106	0.854	5.191				
Extrascleral extension No *vs*. Yes	0.008	0.219	0.071	0.675	0.125	0.392	0.118	1.297
*PVT1* expression High *vs*. Low	0.0003	9.748	2.872	33.080	0.009	12.015	1.854	77.876
*PVT1* DNA methylation High *vs*. Low	0.0006	0.148	0.050	0.443	0.751	1.317	0.240	7.231

### *PVT1* expression is not associated with OS in skin cutaneous melanoma

**Fig 3:**
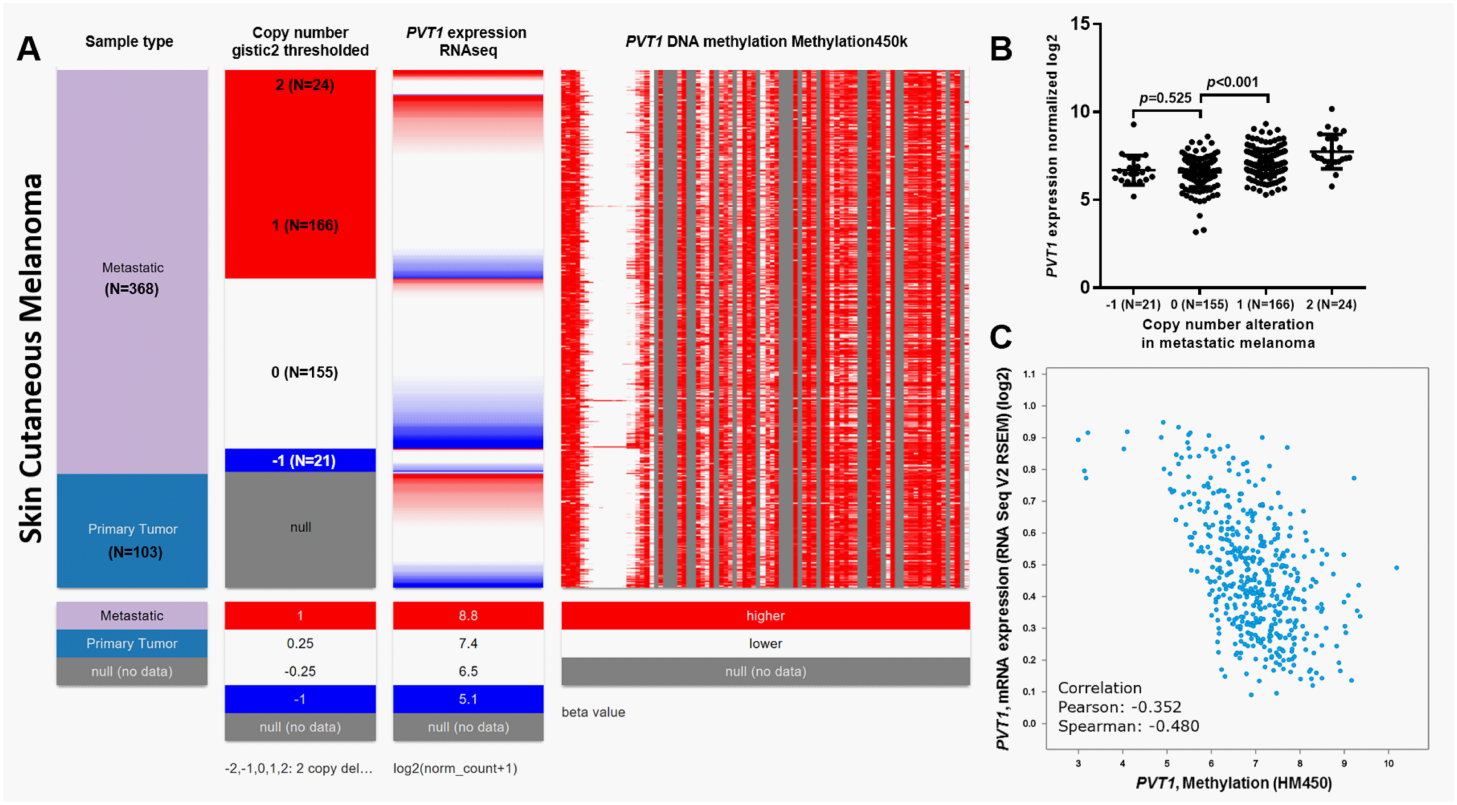
The association between DNA amplification/methylation and *PVT1* expression in skin cutaneous melanoma. **A**. Heatmap of *PVT1* copy number alterations, *PVT1* RNA expression and *PVT1* DNA methylation in 368 metastatic skin melanoma cases and in 103 primary skin melanoma cases. **B**. Bar chart of *PVT1* expression in high-amplification (2), amplification (1), no change (0) and heterozygous loss (-1) groups. **C**. Regression analysis of the correlation between *PVT1* RNA expression and DNA methylation.

**Fig 4:**
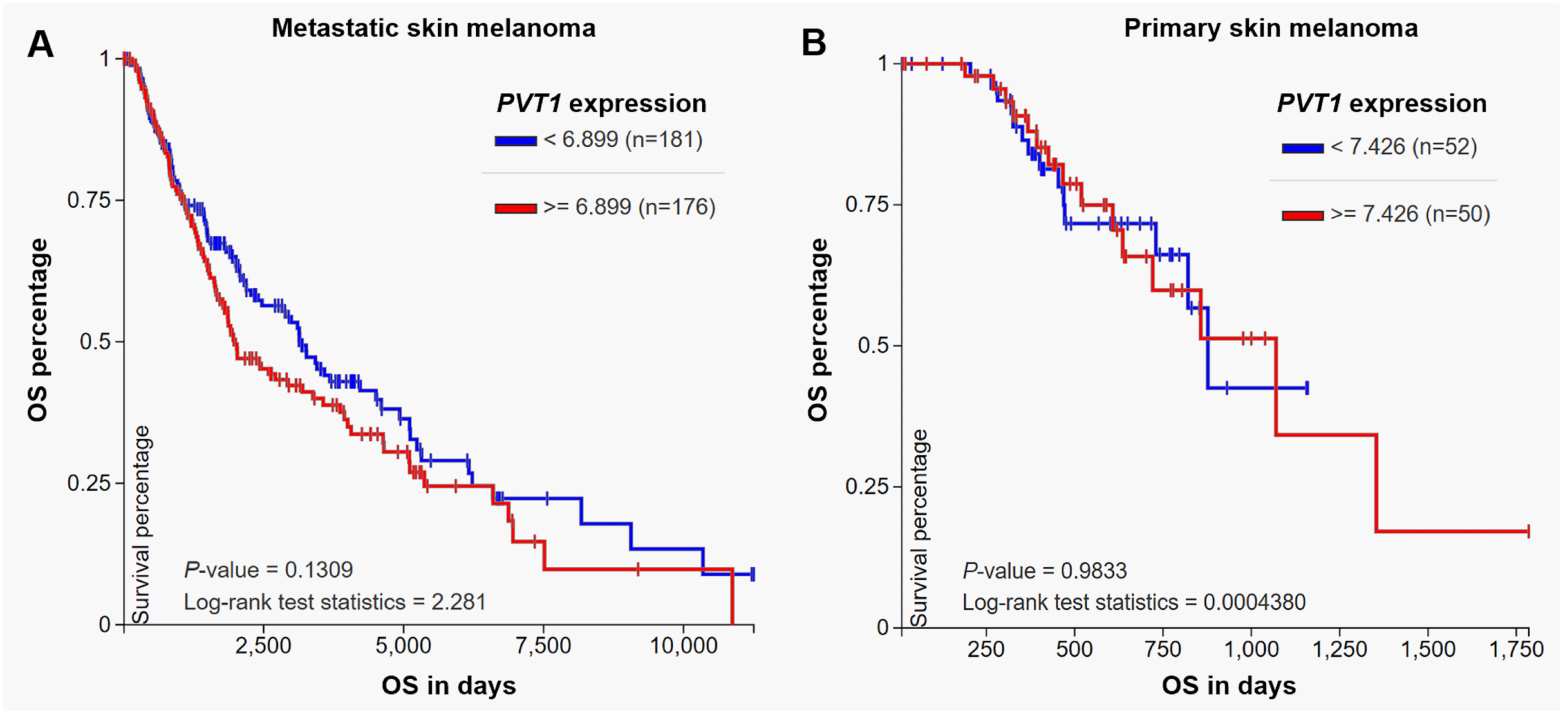
Kaplan-Meier curves of OS in metastatic skin melanoma (A) and in primary skin melanoma (B). Patients were divided into two groups according to the median *PVT1* expression.

To further verify the specificity of the prognostic value of *PVT1* in uveal melanoma, we also examined its expression profile and the association with OS in patients with primary skin cutaneous melanoma in TCGA. DNA copy number alteration data (N = 366) were only available in patients with metastatic skin melanoma (N = 368). *PVT1* DNA amplification was less common in metastatic skin melanoma (190/366, 51.9%) than in uveal melanoma (61/80, 76.3%) (Fig 3A). 21 cases even had *PVT1* heterozygous loss (Fig 3A). Elevated *PVT1* transcription was also observed in DNA amplification group (Fig 3B). In comparison, heterozygous loss did not necessarily result in *PVT1* decrease (Fig 3B). DNA methylation was weakly and negatively correlated with PVT1 expression in skin melanoma (Pearson's r = -0.352, Spearman’s r = -0.480) (Fig 3C). 357 metastatic patients and 102 primary patients with intact OS data were included in Table 3. According to the best cut-off of *PVT1* expression, these patients were divided into high *PVT1* (N = 230, which include 163 metastatic cases and 67 primary cases) and low *PVT1* (N = 229, which include 194 metastatic cases and 35 primary cases) expression groups. The association between *PVT1* expression and the clinicopathological parameters in this group of patients was summarized in Table 3. The high *PVT1* expression group had a higher ratio of primary tumor (67/230) compared with the low *PVT1* expression group (35/229) (*p* = 0.0004) (Table 3). No significant difference in the other parameters was observed between the high and low *PVT1* expression groups (Table 3). Log-rank test of OS curves indicated that *PVT1* expression was not related to OS, no matter in metastatic melanoma (*p* = 0.13, Fig 4A) or in primary melanoma (*p* = 0.98, Fig 4B).

**Table 3:** The association between *PVT1* expression and the clinicopathological parameters in patients with primary skin cutaneous melanoma in TCGA.

Parameters		*PVT1* expression RNAseq		χ^2^	*p*-value
		High (N = 230)	Low (N = 229)		
**Age (Mean ± SD)**		58.32±12.15	57.88±16.31		0.76
**Sample type**	Metastatic	163	194	12.73	0.0004
	Primary Tumor	67	35		
**Gender**	Female	79	95	2.48	0.12
	Male	151	134		
**pathological T**	T0	16	7	3.99	0.26
	Tis	3	4		
	T1/T2	56	62		
	T3/T4	119	121		
	TX/null	36	35		
**Pathological N**	N0	115	114	0.016	0.90
	N1+	90	87		
	NX/null	25	28		
**Pathological M**	M0	205	204	0.15	0.70
	M1+	13	11		
	Null	12	14		
**Pathological stages**	0	2	4	0.86	0.65
	I/II	117	108		
	III/IV	97	95		
	Null	14	22		
**Living Status**	Living	120	117	0.054	0.82
	Dead	110	112		

TX: Primary tumor cannot be assessed; T0: No evidence of primary tumor; NX: Nearby (regional) lymph nodes cannot be assessed; N1+: N1/N2/N3; M1+: M1a/M1b/M1c; null: no data.

## Discussion

*PVT1* has been demonstrated as an oncogenic lncRNA that is usually upregulated in cancer tissues compared with normal tissues. It exerts a carcinogenetic effect via various epigenetic mechanisms such as regulating transcription activity and via acting as miRNAs sponges. For example, it can promote non-small cell lung cancer cell proliferation by recruiting EZH2 to the large tumor suppressor kinase 2 (LATS2) promoter and represses LATS2 transcription [11]. It suppresses miR-146a expression by inducing the methylation of CpG island in its promoter in prostate cancer cells [16]. It can also sponge miR-186 in gastric cancer cells [17], miR-448 in pancreatic cancer cells [18], miR-26b in melanoma cancer cells [13], miR-203 in esophageal squamous cell carcinoma cells [19], thereby contributing to malignant behaviors of these cancers, such as enhanced proliferation, migration, and metastasis.

One recent meta-analysis based on 1,443 patients from 15 previous studies found that increased *PVT1* expression was significantly associated with positive lymph node metastasis, positive distant metastasis, advanced tumor-node-metastasis stage and poor differentiation grade, but was not related to tumor size in some cancers [20]. In this study, we found that high *PVT1* expression was associated with a higher proportion of epithelioid cell dominant disease (a more malignant histological subtype than spindle cell dominant disease) and more cases of extrascleral extension (a risk factor for metastasis), suggesting that high *PVT1* expression may confer some malignant phenotypes to uveal melanoma. However, we did not find a significant difference in tumor size and thickness between the high and low *PVT1* expression groups, which were in consistent with the findings in the meta-analysis.

The mechanisms of *PVT1* dysregulation in these cancers are quite complex and far from being fully understood. *PVT1* locates at 8q24 in the human genome, a region that is usually amplified in some cancers [8, 21]. In gastric cancer, FOXM1 can bind to the promoter region of *PVT1* and enhance its transcription [22]. Upregulation of SOX2 can activate *PVT1* expression in breast cancer cells via binding to its promoter and promote breast cancer cell growth and invasion [23]. These findings indicate that dysregulated *PVT1* may be caused by both genetic and epigenetic alterations. By examining copy number alterations in TCGA-UVM, we found that 61 out of 80 cases (76.3%) of primary uveal melanoma had *PVT1* amplification. In addition, the amplification was associated with significantly higher *PVT1* RNA expression. These findings supported our hypothesis that genetic amplification is a mechanism of aberrant *PVT1* expression in uveal melanoma. Interestingly, by analyzing the DNA methylation status of *PVT1*, we observed a strong negative correlation between *PVT1* expression and its methylation (Pearson's r = -0.712, Spearman’s r = -0.806), suggesting that DNA methylation status can also influence *PVT1* expression in uveal melanoma. In addition, we also observed different levels of copy number alterations and methylation status between uveal melanoma and skin cutaneous melanoma, which indicate that *PVT1* dysregulation might be cancer-specific.

Several previous studies also observed the promising prognostic value of *PVT1* in multiple types of cancer. In gastrointestinal cancers, elevated *PVT1* expression was significantly related to poor OS, DFS, disease-specific survival (DSS) and relapse-free survival (RFS) [10, 24]. Its overexpression also serves as an independent prognostic indicator for the OS of patients with small cell lung cancer [25]. By performing univariate and multivariate analysis, we found that high *PVT1* expression was an independent predictor of poor OS in patients with uveal melanoma (HR: 12.015, 95%CI: 1.854–77.876, *p* = 0.009). In comparison, although it acts as an oncogene in skin melanoma, it had no prognostic value in terms of OS. Therefore, the expression of *PVT1* might serve as a valuable and specific prognostic biomarker in uveal melanoma.

This study also has some limitations. The most important one is that some patients’ characteristics (such as pigmentation) and treatment information was not recorded in the database. In fact, pigmentation has a critical role in melanoma biology [26]. It affects melanoma patients’ survival, radiotherapy, chemotherapy and immune therapy [27–30]. Melanogenesis could simulate HIF-1α expression, thereby conferring malignant behaviors of melanoma cells [30, 31]. Inhibition of melanogenesis might enhance the efficacy of radiotherapy and chemotherapy in advanced melanomas [32, 33]. Another limitation is the relatively small sample size in the database (N = 80). In addition, due to insufficient data, we were unable to assess the association between *PVT1* and DFS among the patients. Therefore, it is meaningful to assess its independent prognostic value in uveal melanoma in a larger cohort in the future.

## Conclusion

Aberrant *PVT1* expression is associated with malignant behaviors of uveal melanoma and might independently predict poor OS.
